# Differential *Plasmodium falciparum* infection of *Anopheles gambiae s.s*. molecular and chromosomal forms in Mali

**DOI:** 10.1186/1475-2875-11-133

**Published:** 2012-04-27

**Authors:** Rebecca T Trout Fryxell, Catelyn C Nieman, Abdrahamane Fofana, Yoosook Lee, Sekou F Traoré, Anthony J Cornel, Shirley Luckhart, Gregory C Lanzaro

**Affiliations:** 1 Department of Entomology and Plant Pathology, University of Tennessee, Knoxville, TN, 37996, USA; 2 Vector Genetics Laboratory, Department of Pathology, Microbiology and Immunology, School of Veterinary Medicine, University of California – Davis, Davis, CA, 95616, USA; 3 Malaria Research and Training Center, University of Bamako, Bamako, Mali; 4 Department of Entomology, Mosquito Control Research Laboratory, University of California – Davis, Davis, CA, 95616, USA; 5 Department of Medical Microbiology and Immunology, School of Medicine, University of California – Davis, Davis, CA, 95616, USA

**Keywords:** *Anopheles gambiae*, Mali, Malaria, *Plasmodium falciparum*, Molecular form, Chromosomal form

## Abstract

**Background:**

*Anopheles gambiae sensu stricto* (*s.s.*) is a primary vector of *Plasmodium falciparum* in sub-Saharan Africa. Although some physiological differences among molecular and chromosomal forms of this species have been demonstrated, the relative susceptibility to malaria parasite infection among them has not been unequivocally shown. The objective of this study was to investigate *P. falciparum* circumsporozoite protein infection (CSP) positivity among *An. gambiae s.s.* chromosomal and molecular forms.

**Methods:**

Wild *An. gambiae* from two sites Kela (n = 464) and Sidarebougou (n = 266) in Mali were screened for the presence of *P. falciparum* CSP using an enzyme-linked immunosorbent assay (ELISA). Samples were then identified to molecular form using multiple PCR diagnostics (n = 713) and chromosomal form using chromosomal karyotyping (n = 419).

**Results:**

Of 730 *An. gambiae sensu lato* (*s.l.*) mosquitoes, 89 (12.2%) were CSP ELISA positive. The percentage of positive mosquitoes varied by site: 52 (11.2%) in Kela and 37 (13.9%) in Sidarebougou. Eighty-seven of the positive mosquitoes were identified to molecular form and they consisted of nine *Anopheles arabiensis* (21.4%)*,* 46 S (10.9%), 31 M (12.8%), and one MS hybrid (14.3%). Sixty of the positive mosquitoes were identified to chromosomal form and they consisted of five *An. arabiensis* (20.0%)*,* 21 Savanna (15.1%), 21 Mopti (30.4%), 11 Bamako (9.2%), and two hybrids (20.0%).

**Discussion:**

In this collection, the prevalence of *P. falciparum* infection in the M form was equivalent to infection in the S form (no molecular form differential infection). There was a significant differential infection by chromosomal form such that, *P. falciparum* infection was more prevalent in the Mopti chromosomal forms than in the Bamako or Savanna forms; the Mopti form was also the most underrepresented in the collection. Continued research on the differential *P. falciparum* infection of *An. gambiae s.s.* chromosomal and molecular forms may suggest that *Plasmodium – An. gambiae* interactions play a role in malaria transmission.

## Background

Several members of the *Anopheles gambiae* complex are vectors of human malaria parasites, including *Plasmodium falciparum*, the species of greatest public health importance in Sub-Saharan Africa. The *An. gambiae* complex consists of at least seven member species and subspecies [1-3]. *Anopheles gambiae sensu stricto* (*s.s.*) is further divided into chromosomal and molecular forms [3]. Molecular studies of the ribosomal DNA region on the X chromosome revealed a fixed difference between populations of *An. gambiae* that is the basis of the sub-division into the M and S molecular forms [4]. The M and S forms are assortatively mating discreet forms [5-7] that are hypothesized to be undergoing ecotypic speciation due to different larval habitat adaptation [8-10]. *Anopheles gambiae* are also divided into chromosomal forms based on the arrangements of 5 paracentric inversions on 2R (j, b,c, u and d) and one on 2 L (a), which define Mopti from Savanna from Bamako forms [3,11]. In Mali, the M form generally associates with the Mopti chromosomal form and the S form with the Savanna and Bamako forms [12]. In some locations in Africa, including Mali, the presence of significant deficiencies in certain inversion heterozygotes suggests there are barriers to gene flow among the chromosomal forms [11,13-16].

Genetic polymorphisms within *An. gambiae* have been associated with adaptation to different local environments [17-21]. For example, a number of genetic polymorphisms are associated with tolerance of arid conditions [22]. There is also significant interest in genotypic variation associated with malaria parasite infectivity. *Plasmodium falciparum* circumsporozoite protein (CSP) positivity in *An. gambiae* was significantly greater in mosquitoes with isozyme allotypes *Mpi*^*130/130*^ and *Acp*^*110/100*^[23]. In addition, 2La/a individuals were nearly twice as likely to be positive than 2La^-^/a^-^[24]. Quantitative trait loci associated with resistance to parasite development have also been identified on chromosome 2 L [25]. Chromosome 2 L includes *APL1*[26], and chromosome 3 L includes *TEP1r*[27] both genes are thought to have anti-parasitic properties. Genome-wide transcriptome analyses of *An. gambiae* have identified transcript expression patterns that are significantly associated with malaria parasite and bacterial infection [28-31]. Further, some transcription patterns and infection-associated sequence polymorphisms have been associated with *An. gambiae* laboratory and field-collected M and S molecular forms [32,33]. Three single nucleotide polymorphisms (SNPs) in immune signaling genes of Malian *An. gambiae* were significantly associated with natural *P. falciparum* infection [33]. These SNPs are predicted to alter the structure and function of the encoded proteins and, therefore, alter refractoriness and susceptibility to *P. falciparum* infection [33]. Additionally, population-specific SNPs associated with either the M or S molecular forms were associated with *P. falciparum* infection, indicating potential differential immune responses of the two molecular forms to parasite infection [33]. Recently, a cryptic subgroup of *An. gambiae* was identified in the Sudan-Savanna zone as susceptible to *P. falciparum*[34].

*Reports of genetic, proteomic, and genomic differences in An. gambiae s.s. suggest, by extension, that susceptibility to P. falciparum* infection varies among different molecular forms and populations of *An. gambiae*[25,27,33,35]*.* This study examined in more detail the hypothesis that *P. falciparum* infection prevalence of *An. gambiae s.s*. differs among molecular and chromosomal forms in Mali. Natural *P. falciparum* infection levels were compared among *An. gambiae* molecular and chromosomal forms in two villages in Mali where these forms occur in sympatry. Inversion-specific correlations between *P. falciparum* infection and standard, heterozygous, and homozygous chromosomal arrangements [3] were also investigated.

## Methods

### Mosquito collections

Adult *An. gambiae* mosquitoes were collected in October 2009 from the villages of Kela (11.88683 N, -8.44744 W), and Sidarebougou (11.4568 N,–5.7323 W) joined with Kolayerebougou (11.4563 N,–5.746 W) in Mali. Resting mosquitoes were collected in the morning via mouth aspirators from inside homes. Mosquitoes were held in cups until they had reached the half-gravid stage. Mosquitoes morphologically identified as *An. gambiae s.l*. were dissected and separated into head/thorax for *P. falciparum* CSP ELISA, abdomen/wings/legs for molecular form identification via polymerase chain reaction (PCR), and ovaries for chromosomal form identification via karyotyping. Head/thorax samples were stored in 100% ethanol, while abdomens/legs/wings were stored in 70% ethanol and half-gravid extracted ovaries were stored in modified Carnoy’s solution (3:1 ethanol to glacial acetic acid).

### ELISA identification of *P. falciparum* infection

Lysates of head/thorax samples were assayed using a P*. falciparum* CSP ELISA [36,37] according to protocols provided by the Centers for Disease Control and Prevention (Atlanta, Georgia, USA) to identify the sporozoite stage (not gametocyte) and that the *P. falciparum* protozoan had disseminated across the midgut. The head and thorax, stored in 100% ethanol, were dried prior to tissue lysis. For each ELISA plate, a minimum of two colony-reared *An. gambiae* mosquitoes (e.g., negative controls) and serial dilutions of *P. falciparum* monoclonal antibodies (i.e., sensitivity positive controls) were used. The positive control *P. falciparum* CSP was serially diluted (i.e., 100 pg to 1.5 pg of antigen per 50 μl of blocking buffer) to quantify CSP in field-collected mosquitoes. Samples with absorbance values greater than three times the standard deviations from the mean of the negative control samples on each ELISA plate were designated as “positive” for *P. falciparum* infection [38]. CSP ELISAs were conducted instead of PCR for molecular detection of *P. falciparum* to ensure the protozoan had disseminated the midgut and that the protozoan was in the ‘infective’ sporozoite phase.

### Identification of species and molecular forms

Abdomen, legs and wings stored in ethanol were ground using a TissueLyser (Qiagen, Valencia, CA, USA), after which DNA was extracted using the BioSprint 96 Bloodkit and automated workstation (Qiagen, Valencia CA, USA). Mosquitoes morphologically identified as *An. gambiae s.l.* were identified to species [39] and *An. gambiae* molecular form identifications were performed on each mosquito [40-42].

### Cytogenetics

Polytene chromosome spreads were prepared from ovarian nurse cells [43], except that spreads were not stained with lacto-orcein prior to examination. Chromosome banding patterns were visualized using an Olympus BX-50 phase contrast microscope. Species identification and paracentric inversion scoring were accomplished using the polytene chromosome maps for *An. gambiae* complex and chromosomal forms [3,11].

### Statistical analyses

Summary statistics, relative abundance of forms, Fisher’s exact tests and two-tailed T-tests were performed in Excel 2007 to determine differences within populations [44]. Where appropriate, we adjusted p-values for multiple comparisons using the Bonferroni correction for an α of 0.05. For the molecular form comparisons, five Chi-square comparisons were conducted (form, village, form x site) that generated a significant p-value less than 0.010. Six comparisons were performed with the chromosomal form data (form, village, form x village) and after the Bonferroni correction, Chi-square comparisons were considered significant if the p-value was less than 0.008. Eighteen comparisons were performed with the karyotype data (arrangement, village, arrangement x village) and, after the Bonferroni correction was applied, Chi-square comparisons were considered significant if the p-value was less than 0.003.

## Results

In total, 730 *An. gambiae s.l*. were analysed, of which 42 (5.8%) were identified as *An. arabiensis*[3,39]. Data from Kolayerebougou and Sidarebougou were combined (hereafter Sidarebougou) because they are located within 1.5 km of one another, have similar habitats, and likely represent a single Mendelian population. In Kela, 21.9% (7/25) of *Anopheles arabiensis* were CSP ELISA positive and 20% (2/10) were CSP ELISA positive in Sidarebougou. Seventeen of the mosquitoes were not identified to molecular form or karyotyped, and two were positive, both from Kela. The remaining 671 mosquitoes were identified as *An. gambiae s.s*., of which 78 (11.6%) were *P. falciparum* CSP ELISA positive. Infection prevalence differed for the two collection sites, from 13.9% (35/251) in Sidarebougou and 10.2% (43/420) in Kela, but they were not significantly different (*X*^*2*^ = 2.101, df = 1, *P* = 0.1472).

**Figure 1:**
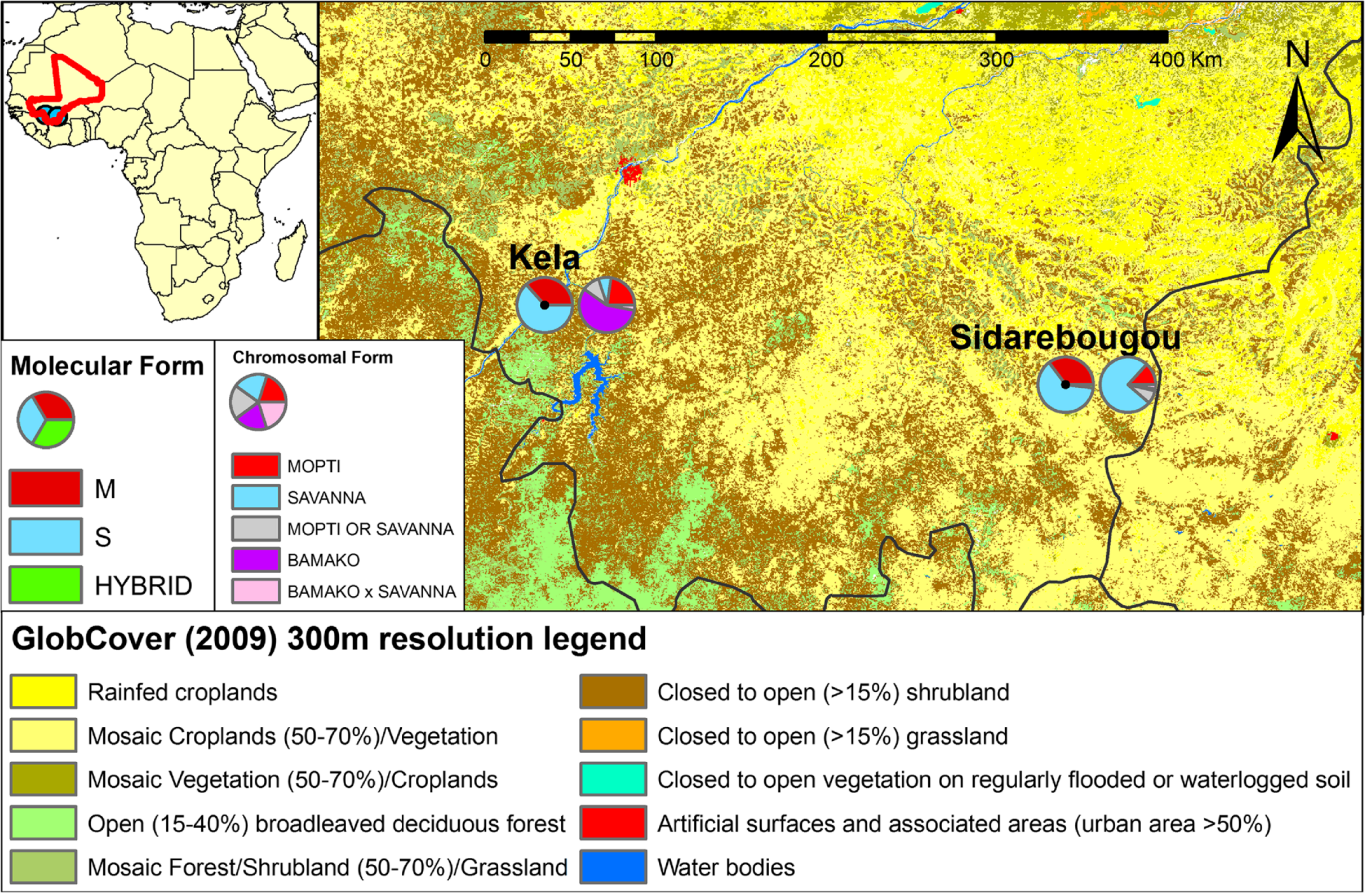
**Molecular (left) and chromosomal (right) form frequencies in*Anopheles gambiae s.s.*collected from Kela and Sidarebougou in western Mali during October 2009.** Mopti/Savanna refers to karyotypes could be classified as either Mopti or Savanna (e.g., bcu heterozygotes). Black point in the middle of the molecular graph identifies the site of the village.

The frequencies of molecular and chromosomal forms of *An. gambiae s.s* are presented in Figure 1. Of the 713 mosquitoes identified, 671 were identified to *An. gambiae s.s*. molecular form (94.1%) and 372 were identified to *An. gambiae s.s*. chromosomal form (52.1%). Both molecular and chromosomal form identifications were performed for 395 mosquitoes of which 370 were *An. gambiae s.s.* and 25 were *An. arabiensis*, separate analyses were conducted for each.

### Molecular form and *P. Falciparum* infection

Infectivity and molecular form association analyses were conducted on 671 *An. gambiae s.s.* This sample was comprised of 62.9% S, 36.1% M and 1.0% M/S molecular form hybrids, but the relative abundance of each molecular form and infection prevalence varied between sites (Figure 1A, Table 1). Within Kela, 63.1% were S, 36.4% were M and 0.5% were M/S hybrids whereas within Sidarebougou 62.5% were S, 35.5% were M and 2.0% were M/S hybrids. Of the 671 mosquitoes identified to the *An. gambiae s.s.* molecular forms 78 (11.6%) were *P. falciparum* CSP ELISA positive, of which, 31 were M, 46 were S, and 1 was a M/S molecular hybrid. Contingency tests on the number of positive to negative samples for each *An. gambiae* molecular form were not significantly different (*X*^*2*^ = 0.595; df = 2; *P* = 0.743: Bonferroni adjusted p- value = 0.0102) (Table 1). Contingency tests revealed that the number of CSP positive and negative S molecular forms (*X*^*2*^ =0.870; df = 1; *P* = 0.351) or M molecular forms (*X*^*2*^ = 1.07; df = 1; *P* = 0.300) were not significantly different at these collection sites. At Sidarebougou (*X*^*2*^ = 0.427; df = 1; *P* = 0.514) and Kela (*X*^*2*^ = 0.178; df = 1; *P* = 0.673), infection prevalence among molecular forms was not significantly different.

**Table 1:** **The M molecular form of*Anopheles gambiae s.s.*was associated with greater*P. falciparum*CSP positivity than were other molecular forms in October 2009**

**Molecular Form**	**No. Screened**	**No. Pos. (% Pos.)**
Kela		
M	153	17 (11.1%)
S	265	26 (9.8%)
M/S	2	0 (0.0%)
Total	420	43 (10.2%)
Sidarebougou		
M	89	14 (15.7%)
S	157	20 (12.7%)
M/S	5	1 (20.0%)
Total	251	35 (13.9%)
Total in Mali		
M	242	31 (12.8%)
S	422	46 (10.9%)
M/S	7	1 (14.3%)
Total	671	78 (11.6%)

### Chromosomal form and *P. Falciparum* infection

Of 372 karyotyped *An. gambiae s.s*., 37.4% were Savanna, 32.3% were Bamako, 18.5% were Mopti, 9.1% were Mopti-Savanna, and 2.7% were Bamako-Savanna. Of the 207 samples karyotyped from Kela, 57.0% were Bamako, 22.7% were Mopti, 7.2% were Savanna, 10.1% were Mopti-Savanna, and 2.9% were Bamako-Savanna (Figure 1B). In Sidarebougou, the frequency of each chromosomal form was 75.2% Savanna, 13.3% Mopti, 7.9% Mopti or Savanna, 2.4% Bamako-Savanna, and 1.2% Bamako (Figure 1B). A total of 55 (14.8%) of the 372 karyotyped *An. gambiae s.s.* were CSP positive (Table 2). Those mosquitoes that were positive for *P. falciparum* CSP were distributed among chromosomal forms as follows: 21 were Savanna, 21 were Mopti, 11 were Bamako, and two were Bamako-Savanna (Table 2). None of the 34 undifferentiated (2R bcu/+ or bc/u or b/cu) Mopti or Savanna forms were positive. Among the three main chromosomal forms, Mopti had the highest CSP positivity (30.4%), followed by Savanna (15.1%) and Bamako (9.2%), and this trend was significant after Bonferroni correction (*X*^*2*^ = 14.8; df = 2; *P* value = 0.001: Bonferroni adjusted *P* value = 0.009) (Table 2). Overall, Mopti chromosomal forms had nearly twice as many positive specimens (compared to negative specimens) than the Savanna or Bamako forms although fewer Mopti specimens were collected (Table 2).

**Table 2:** **The Mopti chromosomal form of*Anopheles gambiae s.s.*was associated with greater*P. falciparum*CSP positivity than were other chromosomal forms or hybrids in October 2009**

**Species**	**No. Screened**	**No. Pos. (% Pos.)**
Kela		
Bamako	118	11 (9.3%)
Savanna	15	5 (33.3%)
Mopti	47	14 (29.8%)
Bamako x Savanna	6	1 (16.7%)
Mopti or Savanna	21	0 (0%)
Total	207	31 (15.0%)
Sidarebougou		
Bamako	2	0 (0.0%)
Savanna	124	16 (12.9%)
Mopti	22	7 (31.8%)
Bamako x Savanna	4	1 (25.0%)
Mopti or Savanna	13	0 (0.0%)
Total	165	24 (14.5%)
Mali		
Bamako	120	11 (9.2%)
Savanna	139	21 (15.1%)
Mopti	69	21 (30.4%)
Bamako x Savanna	10	2 (20.0%)
Mopti or Savanna	34	0 (0.0%)
Total	372	55 (14.8%)

Based on site, there were no significant differences between the number of CSP positive and negative Bamako (*X*^*2*^ = 0.050; df = 1; *P* = 0.651), Savanna (*X*^*2*^ = 4.35; df = 1; *P* = 0.037), or Mopti chromosomal forms (*X*^*2*^ = 0.029; df = 1; *P* = 0.864). Within Kela, Savanna and Mopti chromosomal forms were significantly more likely to be positive than the Bamako form (*X*^*2*^ = 13.4; df = 2; *P* = 0.001: Bonferroni adjusted *P* value = 0.0085; Table 2). There were no significant differences in infection prevalence based on chromosomal forms within Sidarebougou (*X*^*2*^ = 5.47; df = 2; *P* = 0.065; Table 2). The trend that the most prevalent form from each village (Bamako in Kela and Savanna in Sidarebougou) was least likely to be infected, was noted.

### Chromosomal inversions and *P. Falciparum* infection

There were no significant associations of individual inversions and infection status (Table 3). None of the mosquitoes collected had the standard 2La arrangement, and only 16 were heterozygous for the inversion.

**Table 3:** **Chromosomal inversions in*Anopheles gambiae s.s.*were not significantly associated with*P. falciparum*CSP positivity in October 2009 after the Bonferroni correction (*P*** **< 0.003)**

**Inversion**	**No. Positive / No. Screened (% Positive)**		
	**Kela**	**Sidarebougou**	**Total**
	**32 / 215 (14.9%)**	**27 / 179 (15.1%)**	**59 / 335 (15.0%)**
**2La Inversion**			
Standard	0 / 0 (0%)	0 / 0 (0%)	0 / 0 (0%)
Heterozygous	1 / 2 (50%)	1 / 14 (7.1%)	2/ 16 (12.5%)
Inversion	31 / 213 (14.6%	26 / 165 (15.8%)	57 / 378 (15.1%)
Statistic	NA^a^	NA	*X*^*2*^ = 0.080; df = 1; *P* = 0.777
**2Rb Inversion**			
Standard	8 / 83 (9.6%)	1 / 9 (11.1%)	9 / 92 (9.8%)
Heterozygous	14 / 90 (15.6%)	7 / 47 (14.9%)	21 / 137 (15.3%)
Inversion	10 / 42 (23.8%)	19 / 123 (15.4%)	29 / 165 (17.6%)
Statistic	*X*^*2*^ = 4.48; df = 2; *P* = 0.107	*X*^*2*^ = 0.125; df = 2; *P* = 0.939	*X*^*2*^ = 2.84; df = 2; *P* = 0.242
**2Rc Inversion**			
Standard	11 / 46 (23.9%)	19 / 139 (13.7%)	30 / 185 (16.2%)
Heterozygous	9 / 46 (19.6%)	5 / 30 (16.7%)	14 / 76 (18.4%)
Inversion	12 / 123 (9.8%)	3 / 10 (30.0%)	15 / 133 (11.3%)
Statistic	*X*^*2*^ = 6.31; df = 2; *P* = 0.043	*X*^*2*^ = 2.01; df = 2; *P* = 0.366	*X*^*2*^ = 2.36; df = 2; *P* = 0.307
**2Rd Inversion**			
Standard	31 / 210 (14.8%)	24 / 166 (14.5%)	55 / 376 (14.6%)
Heterozygous	1 / 5 (20.0%)	3 / 12 (25.0%)	4 / 17 (23.5%)
Inversion	0 / 0 (0%)	0 / 1 (0.0%)	0 / 1 (0.0%)
Statistic	NA	*X*^*2*^ = 0.967; df = 1; *P* = 0.326	*X*^*2*^ = 1.01; df = 1; *P* = 0.315
**2Rj Inversion**			
Standard	20 / 93(21.5%)	27 / 176 (15.3%)	47 / 269 (17.5%)
Heterozygous	0 / 0 (0.0%)	0 / 1 (0%)	0 / 1 (0.0%)
Inversion	12 / 122 (9.8%)	0 / 2 (0%)	12 / 124 (9.7%)
Statistic	*X*^*2*^ = 5.67; df = 1; *P* = 0.017	NA	*X*^*2*^ = 4.04; df = 1; *P =* 0.044
**2Ru Inversion**			
Standard	9 / 40 (22.5%)	24 / 153 (15.7%)	33 / 193 (17.1%)
Heterozygous	12 / 52 (23.1%)	3 / 24 (12.5%)	15 / 76 (19.7%)
Inversion	11 / 123 (8.9%)	0 / 2 (0%)	11 / 125 (8.8%)
Statistic	*X*^*2*^ = 8.01; df = 2; *P* = 0.018	*X*^*2*^ = 0.163; df = 1; *P* = 0.686	*X*^*2*^ = 5.78; df = 2; *P* = 0.056

^a^ No calculations were conducted because sample size was too small (n < 5).

### Molecular and chromosomal forms and *P. Falciparum* infection

There were 370 *An. gambiae s.s.* identified to both molecular and chromosomal form. Of the 206 typed from Kela and 165 typed from Sidarebougou, 30 and 24 were CSP positive, respectively. The literature suggests that in Mali, most M molecular forms correlate with the Mopti chromosomal form, and the S molecular form correlates with the Bamako and the Savanna chromosomal forms [12] (Table 4). This was largely the case with these data: 57 of 62 M molecular form samples were identified as Mopti chromosomal form and 118 Savanna and 119 Bamako chromosomal forms were identified as S molecular. Two S molecular forms were identified as Mopti chromosomal forms, whereas 19 M molecular forms were identified as Savanna chromosomal forms. Only 21 of 315 individuals (6.7%) did not follow this trend. Of the five MS molecular hybrids, one was identified as a Bamako chromosomal form and four were identified as Savanna chromosomal forms. In Kela, there were 31 M and one S molecular form mosquitoes identified as MoptixSavanna chromosomal hybrids; six were positive. In Sidarebougou, there were 15 M (one positive) and three S (one positive) identified as MoptixSavanna chromosomal hybrids. Discordant associations (e.g., Savanna chromosomal form with M molecular form or S molecular form with Mopti chromosomal) resulted in these discrepancies. The basis for these discrepancies may stem from the molecular form diagnostics overestimating the number of hybrids in the samples [45].

**Table 4:** ***P. falciparum*CSP positivity of*Anopheles gambiae s.s.*identified to both molecular and chromosomal form. Status as M molecular form and Mopti chromosomal form was significantly associated with CSP positivity in October 2009 (*P*** **= 0.001)**

**No. CSP Pos. / No. Screened (% Pos.)**				
**Chromosomal Form**	**Molecular Form**			**Total**
	**M**	**S**	**M/S**	
**Kela**				
**Bamako**	0/0 (0%)	11/117 (9.4%)	0/1 (0%)	11/118 (9.3%)
**Savanna**	2/6 (33.3%)	5/10 (50.0%)	0/0 (0%)	7/16 (43.8%)
**Mopti**	6/38 (15.8%)	0/1 (0.0%)	0/0 (0%)	6/39 (15.4%)
**Total**	8/44 (18.2%)	16/128 (12.5%)	0/1 (0%)	24/173 (13.9%)
**Sidarebougou**				
**Bamako**	0/0 (0.0%)	0/2 (0%)	0/0 (0.0%)	0/2 (0.0%)
**Savanna**	1/13 (7.7%)	15/108 (13.9%)	1/4 (25.0%)	17/125 (13.6%)
**Mopti**	5/19 (26.3%)	0/1 (0%)	0/0 (0.0%)	5/20 (25.0%)
**Total**	6/32 (18.8%)	15/111 (13.5%)	1/4 (25.0%)	22/147 (15.0%)
**Mali**				
**Bamako**	0/0 (0.0%)	11/119 (9.2%)	0/1 (0.0%)	11/120 (9.2%)
**Savanna**	3/19 (15.8%)	20/118 (16.9%)	1/4 (25.0%)	24/141 (17.0%)
**Mopti**	11/57 (19.3%)	0/2 (0.0%)	0/0 (0.0%)	11/59 (18.6%)
**Total**	14/76 (18.4%)	31/239 (13.0%)	1/5 (20.0%)	46/320 (14.4%)

Among mosquitoes identified as Mopti chromosomal and M molecular forms, 15.8% (6/38) and 26.3% (5/19) were CSP positive in Kela and Sidarebougou, respectively. Among mosquitoes identified as Bamako chromosomal and S molecular forms in Kela, 9.4% (11/106) were CSP positive, whereas none (0/2) with this chromosomal and molecular form combination were CSP positive in Sidarebougou. Among mosquitoes identified as Savanna chromosomal and S molecular forms in Kela, 50.0% (5/10) were CSP positive, while 13.9% (15/108) of this form combination were CSP positive in Sidarebougou. These differences were significant in Kela (*X*^*2*^ = 13.4; df = 2; *P* < 0.001), but not in Sidarebougou (*X*^*2*^ = 1.88; df = 1; *P* = 0.170). On average across villages, 19.3% (11/57) of Mopti M forms were CSP positive, 16.9% (20/118) of Savanna S forms were CSP positive, and 9.2% (11/119) Bamako S forms were positive and these patterns were not significant (*X*^2^ =4.32, df = 2, *P* = 0.115).

## Discussion

An average of 12.2% of *An. gambiae s.s.* resting indoors were *P. falciparum* CSP positive from southern Mali in October 2009. Both molecular forms were CSP ELISA positive and there was no differential infection rate among molecular forms. Significantly more Mopti chromosomal forms (30.4%) were positive than were the Savanna (15.1%) and Bamako (9.2%) chromosomal forms. As the Mopti chromosomal form corresponds to M molecular form in Mali in most cases [12], finding that both M molecular and Mopti chromosomal forms were significantly associated with *P. falciparum* infection is not surprising. Site-specific differences in the number of CSP positive chromosomal form infection between Kela and Sidarebougou were also observed. In particular, the most common chromosomal form in each village, Bamako in Kela and Savanna in Sidarebougou, was least likely to be CSP positive.

The insignificant infection prevalence in the M molecular form in southern Mali corroborates with other studies from Cameroon and Senegal that reported no differences in *P. falciparum* infection between M and S molecular forms [6,46]. A recent *P. falciparum* susceptibility assay among *An. gambiae s.s.* molecular forms from Senegal found significantly higher numbers of *P. falciparum* oocysts and sporozoites in the S molecular form than in the M form [47]. This study analysed field-collected specimens of an unknown-age structure that were naturally infected with *P. falciparum*, whereas the Senegal study [47] collected eggs from the field and allowed the surviving adults to feed directly on a membrane with *P. falciparum* to standardize age and potential for infection. These conflicting findings may result from the origin of field collected samples (Mali vs. Senegal), to different techniques (natural vs. artificial infection), or to a differences in the age structure of the samples. Field studies from multiple sites and over multiple sampling periods are necessary to confirm the observed patterns.

Cryptic genetic differences in *An. gambiae s.s.* among sample sites can also limit comparisons among the previous studies [6,46] and the present study. Genetic subdivisions beyond the M and S form designations have been reported and it is possible that differences among these subdivisions include genes associated with differential response to parasite infection. For example, recent studies in Cameroon demonstrated a subdivision in the M molecular form into discrete Forest-M, characterized as M molecular form and Forest chromosomal form (fixed for standard gene arrangement) and Mopti-M populations with typical Mopti karyotypes [12,21]. In analyses using SNPs from immune signaling genes, three genetically distinct *An. gambiae s.s.* populations were observed in Mali: the M molecular form, the S molecular form (S1), and a subdivided S Pimperena form (S2) [33]. Further, SNPs associated with *P. falciparum* infection were differentially distributed among M, S1, and S2 populations [33]. Of interest, data presented here is similar to that reported in Riehle *et al*[34] where a cryptic subgroup of *An. gambiae,* indistinguishable in molecular form but distinguishable via microsatellites amplified from chromosome 3, was susceptible to *P. falciparum*[34].

Differences in the local environment may likewise affect associations between *An. gambiae* forms and *P. falciparum* infection. For example, Dolo *et al*[48] demonstrated that irrigated zones of Mali allowed for constant CSP positivity across seasons along with low human blood feeding and sporozoites indices, whereas in the non-irrigated zones, CSP positivity fluctuated seasonally, being high in the wet season and low in the dry season. Dolo *et al*[48] hypothesized that malaria prevalence in villages adjacent to irrigated rice fields is consistently low in this environment because adult density is inversely related to blood feeding due to high mosquito densities driving villagers to protect themselves with repellants and bed nets [49,50]. The Bamako chromosomal and S molecular forms were dominant in Kela (~3 km to a river), whereas the Savanna chromosomal form and S molecular form predominated in Sidarebougou (~10 km to agriculture fields). Kela is located close to a river that has the ability to flood and create additional oviposition sites not in the dry season likely increasing mosquito densities (11.2% prevalence), whereas in Sidarebougou mosquitoes densities are likely dependent on the wet season (13.9% prevalence). These habitat differences could contribute to the genetic variation (and potentially phenotypic variation) observed at different locations and, as Dolo *et al*[48] hypothesized, habitat may play a role in the vector ecology of *An. gambiae.* Collectively, these studies highlight the importance of both genetic and environmental determinants of susceptibility to infection.

There were statistically significant *P. falciparum* differential infection rates among chromosomal forms and trends among chromosome inversions. Mopti had the highest CSP positivity (30.4%), followed by Savanna (15.1%) and Bamako (9.2%) forms. The data presented here indicated that Mopti chromosomal forms (2Rbc/u) were more likely to be positive for *P. falciparum* CSP. A previous study in Kenya identified a significant association between the total number of inversions and a decreased likelihood for *P. falciparum* infection [24]. Data presented here did not show this specific association to be statistically significant, but standard chromosomal arrangements tended to increase the likelihood of being CSP positive. In particular, in Kela where the Bamako form was dominant and least likely to be positive, mosquitoes with standard or heterozygous arrangements were more likely to be CSP positive than mosquitoes homozygous for 2Rjcu and 2Rjbcu (Bamako form) (Table 3).

A number of studies have examined associations of chromosomal polymorphisms with malaria infection. A small region of chromosome 2 L has been associated with infection susceptibility, regardless of *P. falciparum* genotype [25]. Genes within this region encode for melanization or parasite encapsulation [25]. Within the 2La region, the *APL1* gene, which encodes for natural resistance to *P. falciparum,* exhibited extremely low genetic diversity within the M molecular form, but high diversity in the S molecular form that may have arisen from larval infection [26,35]*.* Alternatively, higher diversity at the *APL1* locus in the S molecular form may be associated with a more diverse array of responses to *P. falciparum* and reduced susceptibility to infection. Similar results were identified within the anti-parasite and anti-bacterial gene *TEP1r*[27]. Specifically, *TEP1r* was diverged between the M and S molecular forms and one variant showed a strong association with resistance to infection with a rodent malaria parasite and with *P. falciparum*[27]. Additional studies comparing *An. gambiae* molecular and chromosomal forms with *P. falciparum* infection that incorporate *An. gambiae* speciation, genetic diversity at immune loci (e.g., *TEP1r, APL1, Toll5B*) as well as larger temporal and spatial scales may help to extend findings reported in this study.

## Conclusion

Whilst the correlation between M and Mopti forms was expected, higher infection prevalences in the Mopti chromosomal form have not been demonstrated previously. In general, significant differences in *P. falciparum* infection prevalence at geographic locations with multiple molecular and chromosomal forms are likely due to discrepancies in the relative abundance of forms, genetic diversity in immune signaling genes within different forms, age structure of field collections, and local environmental variations that influence infection and transmission success.

## Competing interests

The authors declare we have no competing interests with the work presented in this manuscript.

## Authors’ contributions

AJC, GCL, SL, and YL conceived the study and designed the experiments. YL, AJC, AF, and SFT conducted the work in Mali. YL, CSN, and RTF conducted the laboratory work. RTF analysed the data and wrote the manuscript. All authors have read and approved the final manuscript.
